# The correlation between the enzymatic saccharification and the multidimensional structure of cellulose changed by different pretreatments

**DOI:** 10.1186/s13068-014-0134-6

**Published:** 2014-09-24

**Authors:** Ting Cui, Jihong Li, Zhipei Yan, Menghui Yu, Shizhong Li

**Affiliations:** Institute of Nuclear and New Energy Technology, Tsinghua University, Beijing, 100084 China; Beijing Engineering Research Centre of Biofuels, Tsinghua University, Beijing, 100084 China

**Keywords:** Crystalline cellulose, Allomorph, Crystallinity, Water contact angle, Lattice spacing, Specific surface area

## Abstract

**Background:**

The bioconversion of cellulose into simple sugars or chemicals has attracted extensive attention in recent decades. The crystal allomorphs of cellulose are key factor affecting cellulose saccharification. However, due to the influence of lignin, hemicelluloses, and different characterization methods in the literature, the effect of cellulose allomorphs on cellulose saccharification is still unresolved. Thus, a systematic research on the effect of different cellulose allomorphs on enzymatic saccharification was required.

**Results:**

Multiple approaches, including the use of ionic liquid (IL), ethylenediamine (EDA), glycerol, and sodium hydroxide, were used to pretreat α-cellulose in this work. The properties of the obtained cellulose (crystallinity, lattice spacing, specific surface area, and wettability) were characterized by X-ray diffraction, Brunauer, Emmett, and Teller (BET) specific surface area analysis, and water contact angle analysis, respectively. The distance of the lattice spacing of cellulose III was longer than that of other cellulose samples. The crystallinity and water contact angles of the cellulose samples were ranked in the following order: cellulose treated with IL < cellulose treated with NaOH < cellulose treated with EDA < cellulose without treatment < cellulose treated with glycerol. Cellulose treated with IL, with a crystallinity index value of 20%, was very close to amorphous cellulose. After 72 h hydrolysis, the cellulose conversion ratio ranged from 43% to 99%. Cellulose treated with IL exhibited the best hydrolysis profile, followed by cellulose treated with EDA.

**Conclusion:**

Ionic liquid pretreatment significantly altered the ultrastructure and morphology of cellulose samples, making cellulose much easier for enzymes to digest due to its significantly high amorphous content. However, when the impact of amorphous content was not considered, the allomorph easiest for enzymes to digest was cellulose III, followed by cellulose II, cellulose I_α_, and cellulose I_β_. When the cellulose crystallinity index was similar, the allomorph type was the dominant factor. The amorphous content had a strong positive influence on cellulose digestibility. Water contact angle was also an important factor in evaluating the enzymatic hydrolysis efficiency of cellulose except for cellulose III. A high wettability of cellulose enhanced the enzymatic hydrolysis when the crystal allomorph of all the cellulosic samples was the same.

## Background

Cellulose, the main component of lignocellulose, is the most abundant natural carbohydrate resource on the earth [1]. The bioconversion of cellulose into simple sugars or chemicals has attracted extensive attention for the sustainable development of the human society in recent decades. Cellulose is generally cross-linked with hemicellulose and lignin in the plant cell wall [2]. Hence, it is necessary to disrupt the structure of the original cell wall during the process of cellulose bioconversion. For this reason, most research for bioconversion of lignocellulosic feedstock into simple sugars has focused on increasing the enzyme accessibility of cellulose by the removal of hemicellulose or lignin [3–7]. In order for cellulose to be more conducive to enzymatic saccharification, various pretreatment approaches for cellulosic resources have been developed, such as ball-milling, the use of dilute acid, alkaline treatment, ammonia explosion, and so on [1,3,5,8,9]. However, due to the complex structure of the cell wall, multiple factors (for example, delignification, hemicellulose solubilization, porosity, enzyme accessibility, and cellulose crystallinity) interact with each other, and thus the results drawn from different works in the literature have led to confusing conclusions about the recalcitrance of lignocellulosic feedstock.

Besides the effect of lignin and hemicellulose on cellulose bioconversion, the recalcitrant nature of crystalline cellulose, such as multiple hydrogen bonding and a high degree of crystallinity and hydrophobicity, also contribute to the low saccharification efficiency seen in samples of pure cellulose, Avicel, and cotton fibers [10–12]. Previous research revealed that the modification of the cellulose crystal structure by pretreatments tended to affect the enzymatic hydrolysis of cellulose [13–16]. Chundawat and his co-workers reported that the hydrolysis yield of Avicel increased 1.5- and 2-fold after it was treated with NaOH and NH_3_, respectively [12]. Ciolacu *et al.* also reported that the enzyme saccharification yield of Avicel PH-101 increased from 10% to 62% after it was treated with 17.5% NaOH for 24 h at 15°C, and from 10% to 18% after the samples were soaked in organic amine (100% ethylenediamine, EDA) for 24 h at room temperature [17]. In these works, the particle size of the cellulose samples was changed, and more importantly, the allomorphic form of cellulose was modified. These results indicated that the allomorphic form of cellulose could be a key factor in the improvement of the saccharification of cellulose. Moreover, changing the allomorph of cellulose might be a better alternative and more feasible method to enhance the rate of subsequent enzymatic catalysis, as compared to trying to discover novel microorganisms for the bioconversion process.

The allomorph of native cellulose is cellulose I, including cellulose I_α_ and I_β_ [18]. After pretreatment, cellulose crystallizes into various allomorphs (cellulose I, II, III, and IV) with different packing arrangements [1,19]. Cellulose II is formed when native cellulose regenerates from a dissolved state or is mercerized with alkali. Cellulose I or cellulose II treated with liquid ammonia or certain amines would lead to the formation of cellulose III [1]. Cellulose IV is a disordered form of cellulose I_β_ [19].

Most research conducted on the enzymatic digestibility of cellulose allomorphs only focuses on one form and the related pretreatment [13,20,21]. However, because of the different characterization methods of cellulose properties and hydrolysis conditions utilized in these studies, the data cannot be easily compared to determine the optimal cellulose allomorph for enzymatic bioconversion. We still lack an understanding of the relationship between various cellulose allomorphs and cellulose enzymatic saccharification and of which factors are the most influential in the hydrolysis process.

The main objective of this study was to attain a systematic understanding of the effect of different cellulose allomorphs produced by the various pretreatments on enzymatic saccharification. In this study, we used multiple approaches, including the use of ionic liquid (IL), EDA, glycerol, and sodium hydroxide, to pretreat α-cellulose. The physical and chemical properties (crystallinity index, lattice spacing, specific surface area, and wettability) of the cellulose obtained were characterized by X-ray diffraction (XRD), BET specific surface area analysis, and water contact angle analysis, respectively. The correlation of crystalline cellulose structure and enzymatic hydrolysis was discussed.

## Materials and methods

### Materials

**Figure 1:**
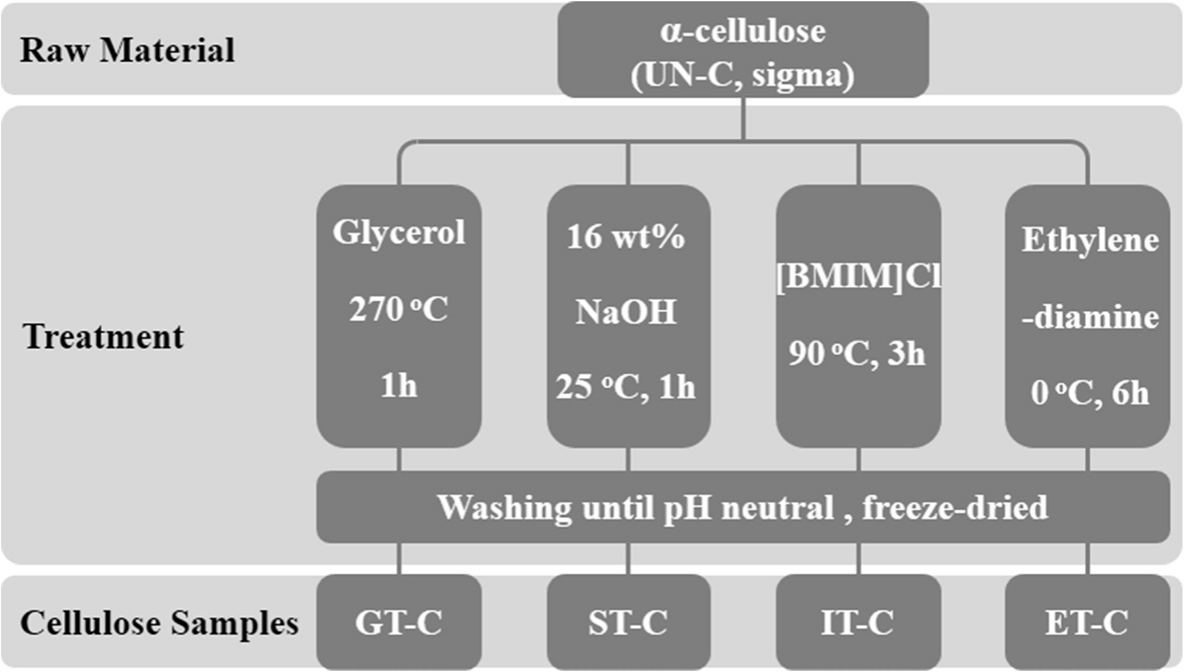
**Different types of crystalline cellulose preparation.**

The cellulose samples were prepared as pictured in Figure 1.

Cellulose I_α_ (untreated cellulose, UN-C): α-cellulose (sigma) was used without further purification.

Cellulose I_β_ (glycerol treated cellulose, GT-C): 0.5 g α-cellulose specimens were inserted in a small glass ampule filled with 5 mL glycerol under nitrogen. The ampule was sealed and heated at 270°C for 1 h. The ampule was then cooled naturally and washed thoroughly with distilled water until it reached neutrality, then lyophilized.

Cellulose II: cellulose II was obtained by two methods.

ST-C (sodium hydroxide treated cellulose): α-cellulose powders were soaked in 16.5% NaOH for 2 h, at 25°C, followed by washing thoroughly with distilled water until neutrality and lyophilization.

IT-C (IL treated cellulose): for the cellulose II samples prepared by IL, α-cellulose was immersed in 1-butyl-3-methylimidazolium chloride ([BMIM]Cl) at 90°C for 3 h, followed by washing thoroughly with distilled water and lyophilization.

Cellulose III (ET-C, EDA treated cellulose): cellulose III was prepared by soaking α-cellulose in EDA for 6 h in an ice-water bath. Then the cellulose-amine complex was washed with anhydrous ethanol until it reached neutrality and then lyophilized.

### Methods

#### Water contact angle

The water contact angles of cellulose substrate were determined using the Kruss Tensiometer K100 (Kruss GmbH, Hamburg, Germany) to determine the tensiometry. The experimental procedures were performed according to the literature [10].

#### X-ray diffraction

The lyophilized samples were scanned on a Bruker D8 Advance diffractometer (Bruker, Germany) using Cu/Kα radiation (1.54 Å) generated at 45 kV and 40 mA, at room temperature. The scan speed was 0.02°s^-1^ with a step size of 0.02°, and the scans were collected from 2θ = 5 to 40°. The crystallinity index (CrI) was calculated using the peak intensity method [22]:

$$ \mathrm{C}\mathrm{r}\mathrm{I}=\frac{\left({\mathrm{I}}_{020}-{\mathrm{I}}_{\mathrm{am}}\right)}{{\mathrm{I}}_{\mathrm{am}}}\ast 100\%, $$

where I_020_ is the intensity at the main peak of the cellulose samples, which usually lies around 22.5°, 21.9°, and 21° for cellulose I, cellulose II, and cellulose III, respectively. I_am_ is the intensity of amorphous cellulose content at 2θ = 18°.

The lattice spacing (d-spacing) was calculated using Bragg’s equation [17]:

$$ \uplambda =2{\mathrm{d}}_{\mathrm{hkl}}\cdotp \sin \uptheta, $$

where d_hkl_ is the lattice spacing of the crystallographic planes, θ is the corresponding Bragg angle, and λ is the X-ray wavelength (0.154 nm).

#### Fourier transform infrared spectroscopy (FTIR) microscopy

Samples were prepared by mixing 2 mg cellulose with 200 mg of spectroscopic grade KBr. The FTIR spectra were recorded using a Nicolet 6700 spectrometer with detector at 0.4 cm^-1^ resolution and 64 scans. To determine the I_α_ fraction in the cellulose samples, the characteristic IR absorption bands at 750 cm^-1^ for I_α_ and 710 cm^-1^ for I_β_ were used to determine the cellulose crystal form, as outlined in a previous report [23]. The IR index for the fraction (*f*_*Iα*_) was calculated, using the band areas for I_α_ (A_750_) and I_β_ (A_710_):

$$ {f}_{I\alpha } = {\mathrm{A}}_{750}/\left({\mathrm{A}}_{750}+{\mathrm{A}}_{710}\right) $$

#### Scanning electron microscopy (SEM)

The morphology of the cellulose samples was analyzed by using a Zeiss Merlin electron microscope (Carl Zeiss Microscopy, Jena, Germany) with a field emission tungsten filament electron gun, operating at 1 kV. Prior to the SEM experiment, the cellulose samples were coated with a 20 to 30 nm C thin film to avoid the charging effect during the testing.

#### Enzymatic hydrolysis

Enzymatic hydrolysis of all cellulose samples was carried out in triplicate, using a rotary shaker at 50°C and 150 rpm by Novozymes CTec3 at 5% solids (w/v). 0.3% (w/v) sodium azide was added to inhibit the growth of microorganisms, while 0.05 M sodium acetate buffer was used to maintain the pH at 4.8. To determine the conversion ratio of cellulose during the enzymatic hydrolysis, the mixture was centrifuged for 5 min at 12,000 rpm to terminate the reaction after 2, 4, 12, 24, 48, and 72 h. The carbohydrate concentration was analyzed by an HPLC instrument (Shimadzu, Kyoto, Japan). The cellulose conversion ratio was calculated according to the follow formula:

$$ \begin{array}{l}\mathrm{Cellulose}\ \mathrm{conversion}\ \mathrm{ratio} = 0.9 \times \mathrm{glucose}\ \mathrm{concentration} \times \mathrm{hydrolysate}\ \mathrm{volume}/\mathrm{the}\ \mathrm{weight}\\ {}\mathrm{of}\ \mathrm{cellulose}\ \mathrm{substrate} \times 100\%\end{array} $$

#### BET specific surface area

Cellulose samples were oven-dried at 80°C for 10 h to minimize structural changes prior to BET analysis with N_2_. The BET specific surface area and pore volume distribution were determined by a Micromeritics Tristar 3020 II analyzer (Micromeritics Instrument Corporation, Norcross, GA, USA) at a relative pressure approximating unity [24].

## Results

### Cellulose allomorphs and crystallinity

**Figure 2:**
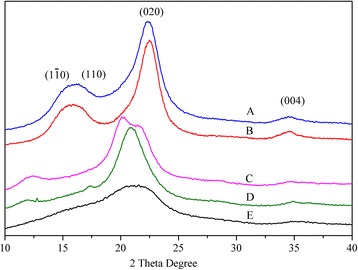
**X-ray diffraction patterns of cellulose samples: (A) UN-C; (B) GT-C; (C) ST-C; (D) ET-C; (E) IT-C.**

XRD patterns of all the cellulose samples are shown in Figure 2. The results were consistent with the data reported by previous research focusing on the allomorphs of celluloses [12,15,17,20]. The cellulose samples treated with glycerol presented characteristic 2θ diffraction planes at 15° (1Ī0), 16° (110), and 23° (020), which are still those of cellulose I. However, the allomorphic change to I_β_ from I_α_ is difficult to determine with diffraction patterns. To further examine the crystalline structure of GT-C, the FTIR spectra of GT-C were recorded. As expected, the content of allomorph I_β_ in the GT-C samples exceeded 55%, demonstrating a partial change to allomorph I_β_ (Table 1). After NaOH or [BMIM]Cl treatment, α-cellulose converted to cellulose II with a doublet appearing at 2θ values (about 20° and 22°) for the 1Ī0 and 020 peak. α-cellulose converted to cellulose III during the EDA treatment, with the position of the 020 peak shifting from a 2θ value of 23° to 21° [6].

**Table 1:** **The I**_**α**_ **fraction in GT-C samples**

**Cellulose sample**	**Cellulose I**_**α**_ **fraction (%)**	**Cellulose I**_**β**_ **fraction (%)**
UN-C	83	17
GT-C	42	58

The CrI of α-cellulose without any further treatment was 61%. The ET-C and ST-C samples exhibited a CrI of 55% and 46%, respectively. After pretreatment, the IT-C samples had a CrI value of 20%, a substantial reduction in the CrI value which is very close to that of amorphous cellulose. In addition, GT-C had the lowest amorphous content, with a 69% CrI value.

### Cellulose wettability

The cellulose wettability was characterized by measuring the cellulose water contact angle using the Kruss K100. The water contact angles of the cellulose samples were ranked in the following order: IT-C < ST-C < ET-C < UN-C < GT-C. The water contact angle values for ET-C and UN-C were 58.4° and 60.7° respectively, indicating no significant wettability difference among substrates with EDA treatment. NaOH treatment produced a slight increase in wettability among cellulose samples measured at a water contact angle value of 47.9°. Compared to the above two treatments, cellulose II prepared by IL pretreatment tended to exhibit a greater capacity of water absorption, with a 29.5° water contact angle value. Cellulose treated by glycerol seemed to be the most hydrophobic compared to other samples, with a water contact angle of 66.0°. It was also unique in that it surpassed the value of untreated α-cellulose. These results suggested that glycerol treatment caused the cellulose samples to be more hydrophobic, while the other three treatments changed the cellulose to be more hydrophilic. The greatest wettability cellulose samples were obtained through IL treatment.

### Cellulose BET specific surface area (SSA)

**Figure 3:**
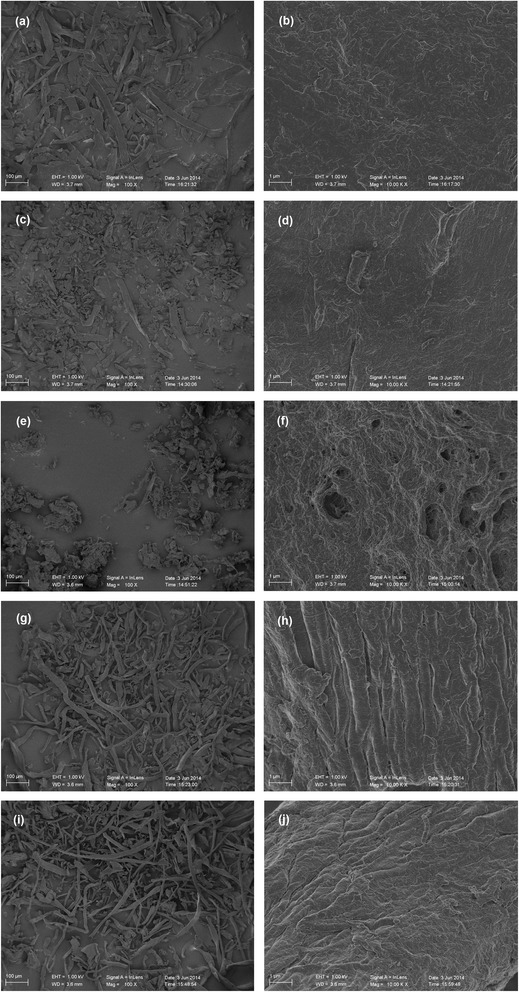
**SEM images of different cellulose samples, UN-C (a, b), GT-C (c, d), IT-C (e, f), ST-C (g, h), and ET-C (i, j).**

The SSA values of cellulose samples were measured using the BET specific surface area method. Both the BET N_2_ absorption of UN-C and GT-C were near zero, and the data was too small to be detected by this method. The BET N_2_ SSA of IT-C (8.47 m^2^/g) significantly exceeded the corresponding values of the other samples. The BET N_2_ SSA of ET-C (0.08 m^2^/g) was larger than that of ST-C (0.06 m^2^/g). As shown in Figure 3, the untreated cellulose samples were intact and had a relatively smooth surface. After glycerol treatment, the surface of the cellulose samples did not show obvious changes, although the average fiber length shortened. With NaOH and EDA pretreatment, the surface of the cellulose samples appeared to be uneven and rough, but still maintained the fiber morphology. After regeneration from IL, the morphology of the cellulose samples was significantly changed. The surface of the IL treated cellulose had a large number of holes and the elongated structure of the fibers had disappeared.

### Enzymatic hydrolysis of different allomorph celluloses

**Figure 4:**
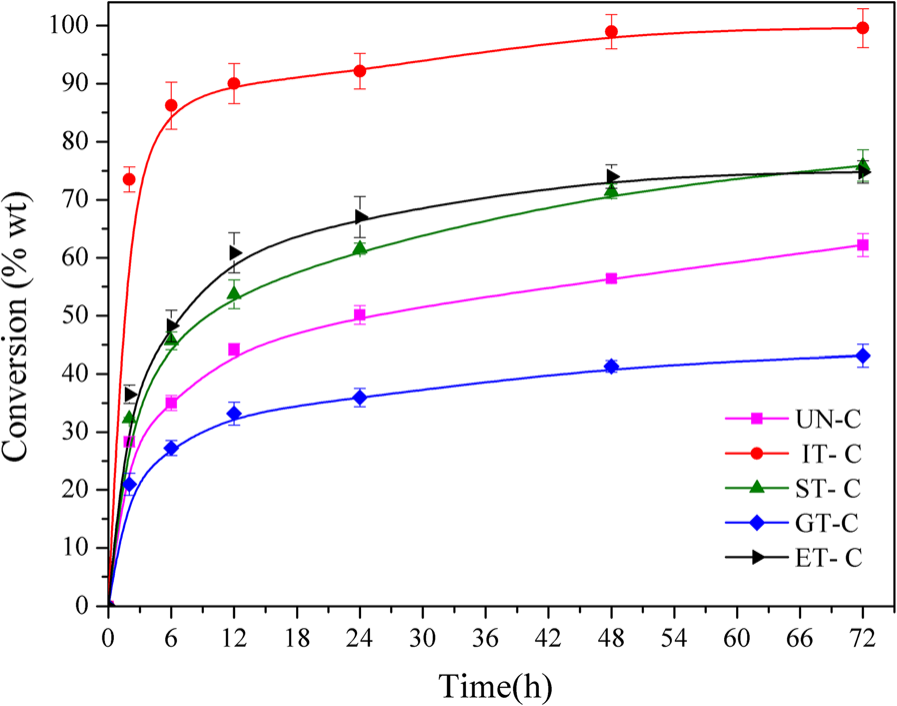
**Enzymatic hydrolysis of cellulose samples.** (The error bars indicate the reproducibility of digestions conducted in triplicate on the cellulose samples).

Samples of different cellulose allomorphs were hydrolyzed for 72 h, and the cellulose conversion ratios ranged from 43% to 99% (Figure 4). IT-C exhibited a notably efficient hydrolysis profile; more than 70% of the substrate was converted into glucose during the first 2 h and the saccharification ratio reached 99% at 72 h. ST-C and ET-C had similar enzymatic rates and the final saccharification ratio at 72 h was about 75%. However, as shown in Figure 4, after being treated by glycerol, the cellulose samples had a much lower glucose yield, which was 30% lower than that of UN-C.

## Discussion

Although the mechanism of cellulose hydrolysis has been studied intensively over the last few decades, it is still unclear because of the complex process of cellulose enzymatic hydrolysis. Most studies have been more concerned with the characterization of the cellulolytic microorganisms, and the biochemical properties of the enzymes produced by them [25,26]. Enhancing the enzymatic hydrolysis rate of lignocellulose substrate and altering the crystalline cellulose features, especially the cellulose allomorphs, is often ignored, because a change of cellulose crystal form does not take place for every treatment. Moreover, changing the cellulose allomorph results in changes to other features of cellulosic substrates, such as the crystallinity index and specific surface area [27]. Thus, a valuable evaluation system to identify the enzymatic digestibility of cellulosic substrates is needed and essential to make the best choice for a pretreatment method.

In this paper, in order to investigate the effects of various cellulose allomorphs on enzyme hydrolysis, several pretreatment methods were utilized to change the crystal form of cellulose to obtain different cellulose allomorph samples. These pretreatments were NaOH mercerization, EDA soaking, glycerol treatment, and IL treatment [1,12,13,15,28–31]. The crystal allomorph of each sample was characterized by XRD or FTIR. GT-C obtained from glycerol treatment was a crystal mixture of 58% I_β_ and 42% I_α_. ST-C and IT-C were allomorph II obtained from NaOH and IL treatment, respectively. ET-C obtained from EDA treatment was cellulose III. Unfortunately, cellulose samples with similar crystallinity index values were not obtained, although we adjusted the pretreatment conditions repeatedly. Furthermore, according to the XRD results, after pretreatments, the CrI of all the cellulose samples decreased except for the sample treated with glycerol. This result indicated that the CrI of cellulose was more sensitive to the pretreatments than the crystal form. In addition, after cellulose dissolved in [BMIM]Cl, most of the crystalline cellulose transformed into an amorphous structure. Among these four pretreatments, the cellulose sample with the lowest CrI was produced during the regeneration process from the dissolved state in IL, most likely because the strong hydrogen bonding network of crystalline cellulose was destroyed by the ionic liquid.

**Figure 5:**
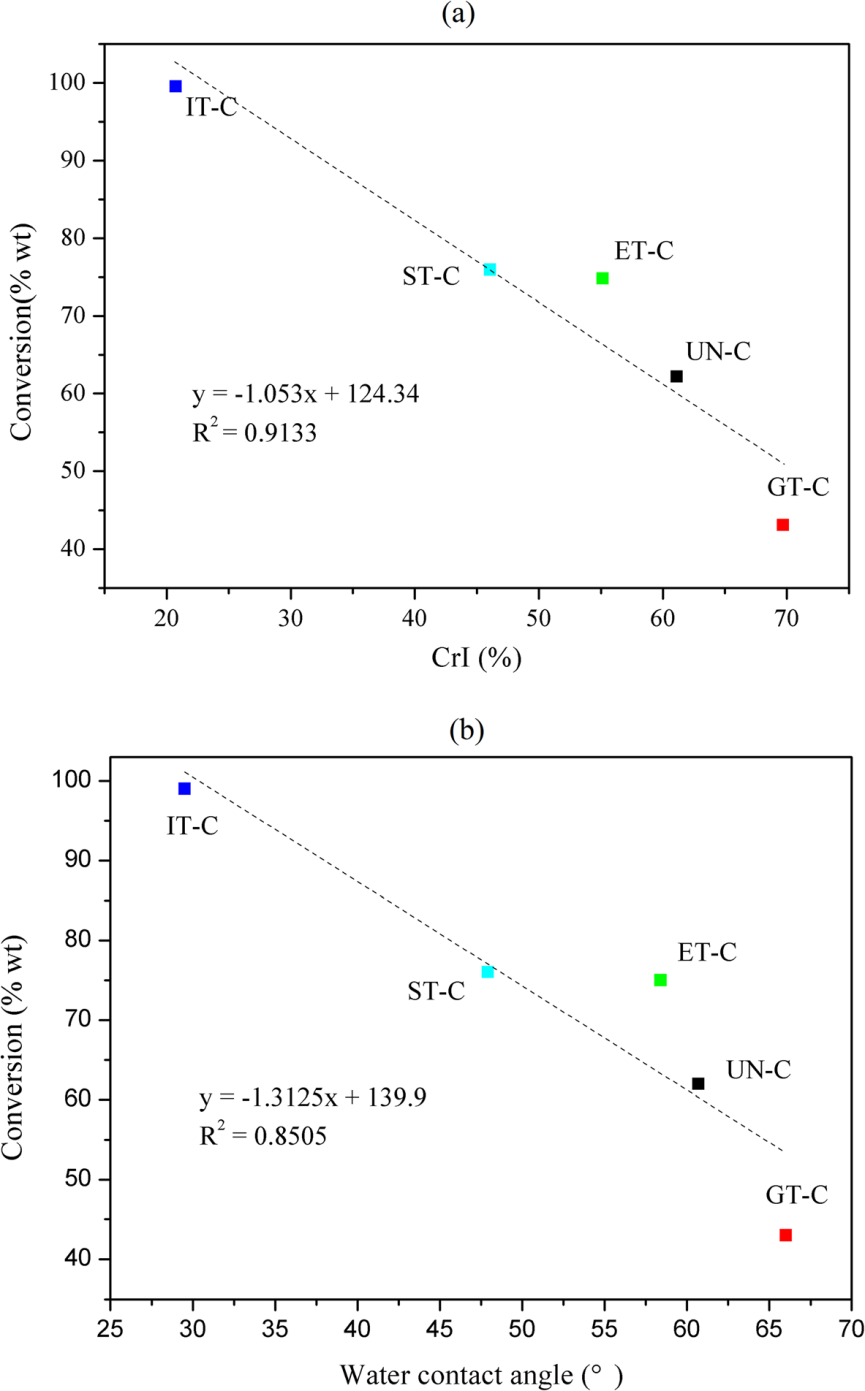
**The correlation between crystallinity index (a), water contact angle (b), and cellulose conversion.**

The CrI of cellulose is a key predictor of the enzymatic hydrolysis rate [11]. The correlation between the CrI of the five samples and cellulose conversion ratio is shown in Figure 5a. As expected, there was a negative correlation between the cellulose conversion ratio and crystallinity, whereas the ET-C sample obtained from the EDA soaking pretreatment had a significant positive deviation. According to the XRD analysis, the allomorph of ET-C was cellulose III. Comparing the CrI of ST-C with IT-C, for the same allomorph, cellulose samples with lower CrI had a much faster saccharification rate. Samples of UN-C and GT-C with a similar crystal allomorph also demonstrated this result. It can be concluded that amorphous content had a strong positive influence on cellulose digestibility. In a previous work, Bertran and Dale reported that most aqueous reagents could only penetrate the amorphous portion of cellulose [32]. Crystallinity and cellulose accessibility were closely related. Additionally, Hall and co-workers reported that, at low degrees of crystallinity, adsorbed enzymes were more active at the same concentration, because a more open cellulose structure would likely prevent enzyme molecules residing on neighboring chains from hindering one another [11]. Cellulose accessibility and cellulase activities were both suggested to be important factors affecting enzymatic hydrolysis rates. Therefore, amorphous content contributed to increased cellulose digestibility.

However, in the case of ET-C, this conclusion did not seem to apply, because its CrI was obviously higher than that of ST-C, but it had a similar cellulose conversion rate as ST-C. In order to discover the reason for this disparity, the specific surface area (SSA), another factor reported to affect the enzymatic hydrolysis of cellulose, was investigated. The SSA of ET-C was slightly higher at 0.08 m^2^/g compared to 0.06 m^2^/g that of the ST-C sample. The small difference in the SSA of ST-C and ET-C seemed insignificant when compared to 8.47 m^2^/g, the value of the IT-C sample. In a previous work, the surface areas of Avicel-based cellulose I and cellulose III were 0.64 and 0.61 m^2^/g, respectively [12]. After IL pretreatment, the surface area of corn stover increased from 0.7 to 15.1 m^2^/g [33]. Therefore, the SSA obviously was not a major factor affecting the hydrolysis of ET-C and ST-C. From the viewpoint of enzyme accessibility, a larger surface area exposure could enhance the cellulose digestibility. Nevertheless, except for IT-C, the other pretreatments had only limited impact on the surface area of the samples.

All three pretreatment reagents, NaOH, EDA, and [BMIM]Cl, could form hydrogen bonds with cellulose, and the stability order of the hydrogen bonds between pretreatment reagent and cellulose was [BMIM]Cl > EDA > NaOH. From the results of cellulose crystallinity and surface area in this study, we demonstrated that the crystalline cellulose structure was stable, and only a strong competitive hydrogen donor-acceptor, such as ionic liquid, could destroy the stable structure and increase the enzyme accessibility of native cellulose. These results could also explain why dilute acid pretreatment, a leading pretreatment technology for lignocellulose feedstock, was unable to loosen the compact structure of cellulose, because dilute sulfuric acid could not form hydrogen bonds with cellulose. This may be the reason that dilute acid pretreatment was not as effective as alkali pretreatment, especially ammonia technology for high crystallinity lignocellulosic feedstock [34–36].

In addition, wettability was reported to be able to predict the enzymatic hydrolysis of cellulose [10]. A higher water contact angle value of the samples indicated more hydrophobicity and inferior wettability. The correlation of wettability and cellulose conversion rate of various allomorphs is shown in Figure 5b. There was a negative correlation between the water contact angle and the cellulose conversion rate except for EC-T. The water absorption value is an indirect measure for internal pore volume and crystallinity of cellulose samples. The increase of cellulose wettability also enabled free enzymes to diffuse more easily from the solution to the cellulose surface [20].

All the characterization results revealed that the EC-T sample had special properties. Although its crystallinity and water contact angle were higher than these of ST-C, its enzymatic hydrolysis efficiency was similar to that of ST-C, which might contribute to the crystal allomorph. Comparing the crystal parameters of various allomorphs shown in Table 2, the distance of lattice 1Ī0 plane of cellulose III was longer than that of cellulose II, and significantly longer than that of cellulose I. The results revealed that the intermolecular force between cellulose chains was weaker with increasing distance. In addition, the enhanced enzymatic digestibility of cellulose III was reported to be related to the “amorphous-like” nature of its surface chains [12].

**Table 2:** **The lattice spacing (d-spacing) of cellulose samples**

**Sample**	**d-Spacing (A)**		
	**(1Ī0)**	**(110)**	**(020)**
UN-C (Cellulose I_α_)	5.45	5.45	3.98
GT-C (Cellulose I_β_)	5.48	5.48	3.95
IT-C (Cellulose II)	5.91	4.11	4.11
ST-C (Cellulose II)	7.11	4.40	4.13
ET-C (Cellulose III)	7.37	4.26	4.26

According to the integrated experimental results (Table 3), the most digestible enzymatic hydrolysis allomorph was amorphous cellulose, followed by cellulose III, cellulose II, cellulose I_α_, and cellulose I_β_. When the cellulose crystallinity index was similar, the allomorph type was the dominant factor. Amorphous content had a strong positive influence on cellulose digestibility. Water contact angle was also an important factor in the evaluation of the enzymatic hydrolysis efficiency of cellulose, except in the case of cellulose III.

**Table 3:** **Cellulose structure and enzymatic digestibility**

**Pretreatment**	**Allomorph**	**CrI (%)**	**Water contact angle** ^**a**^ **(** ^**o**^ **)**	**Surface area (m** ^**2**^ **/g)**	**Cellulose conversion** ^**b**^ **(%)**
[BMIM]Cl	II	20	29.5 ± 0.6	8.47	99 ± 4
EDA	III	55	58.4 ± 0.9	0.08	74 ± 2
NaOH	II	46	47.9 ± 0.4	0.06	76 ± 3
No treatment	I_α_	61	60.7 ± 0.8	-	62 ± 2
Glycerol	I_β_	69	66.0 ± 0.7	-	43 ± 2

^a^The reproducibility of cellulose water contact angle was conducted in triplicate.

^b^The reproducibility of cellulose conversion was conducted in triplicate.

## Conclusion

In this paper, four types of cellulose allomorphs were prepared. Ionic liquid pretreatment significantly altered the ultrastructure and morphology of cellulose samples, making cellulose much easier for enzymes to digest due to its significantly high amorphous content. However, when the impact of amorphous content was not considered, the allomorph easiest for enzymes to digest was cellulose III, followed by cellulose II, cellulose I_α_, and cellulose I_β_. When the cellulose crystallinity index was similar, the allomorph type was the dominant factor. Amorphous content had a strong positive influence on cellulose digestibility. Water contact angle was also an important factor for evaluating the enzymatic hydrolysis efficiency of cellulose except for cellulose III. High wettability of cellulose enhanced the enzymatic hydrolysis when the crystal allomorph of cellulosic samples was the same.

## Abbreviations

BET: Brunauer-Emmett-Teller
[BMIM]Cl: 1-butyl-3-methylimidazolium chloride
CrI: crystallinity index
EDA: ethylenediamine
ET-C: ethylenediamine treated cellulose
FTIR: Fourier transform infrared spectroscopy
GT-C: glycerol treated cellulose
HPLC: high performance liquid chromatography
IL: ionic liquid
IR: infrared spectroscopy
IT-C: ionic liquid treated cellulose
SEM: scanning electron microscopy
SSA: specific surface area
ST-C: sodium hydroxide treated cellulose
UN-C: untreated cellulose
XRD: X-ray diffraction
